# Unique Features of the Human Brainstem and Cerebellum

**DOI:** 10.3389/fnhum.2014.00202

**Published:** 2014-04-07

**Authors:** Joan S. Baizer

**Affiliations:** ^1^Department of Physiology and Biophysics, School of Medicine and Biomedical Sciences, University at Buffalo, Buffalo, NY, USA

**Keywords:** medulla, dentate nucleus, vestibular nuclei, nonphosphorylated neurofilament protein, cerebellar cortex, saccadic eye movements, inferior olive, calcium-binding proteins

## Abstract

The cerebral cortex is greatly expanded in the human brain. There is a parallel expansion of the cerebellum, which is interconnected with the cerebral cortex. We have asked if there are accompanying changes in the organization of pre-cerebellar brainstem structures. We have examined the cytoarchitectonic and neurochemical organization of the human medulla and pons. We studied human cases from the Witelson Normal Brain Collection, analyzing Nissl sections and sections processed for immunohistochemistry for multiple markers including the calcium-binding proteins calbindin, calretinin, and parvalbumin, non-phosphorylated neurofilament protein, and the synthetic enzyme for nitric oxide, nitric oxide synthase. We have also compared the neurochemical organization of the human brainstem to that of several other species including the chimpanzee, macaque and squirrel monkey, cat, and rodent, again using Nissl staining and immunohistochemistry. We found that there are major differences in the human brainstem, ranging from relatively subtle differences in the neurochemical organization of structures found in each of the species studied to the emergence of altogether new structures in the human brainstem. Two aspects of human cortical organization, individual differences and left–right asymmetry, are also seen in the brainstem (principal nucleus of the inferior olive) and the cerebellum (the dentate nucleus). We suggest that uniquely human motor and cognitive abilities derive from changes at all levels of the central nervous system, including the cerebellum and brainstem, and not just the cerebral cortex.

## Introduction

**Figure 1:**
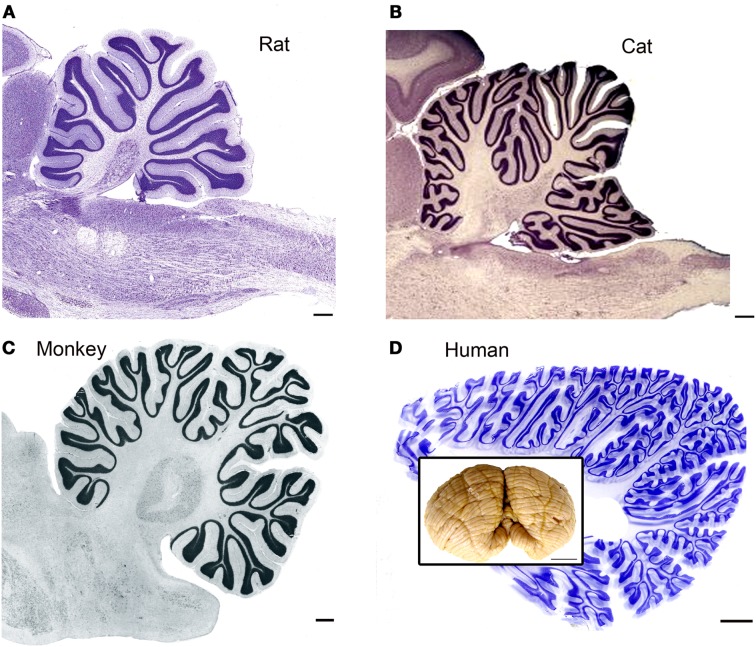
**The cerebellar cortex in four different species**. **(A)** Cresyl violet (CV) stained parasagittal section through the cerebellum and brainstem of a rat. Scale bar 2 mm. The image is from BrainMaps.org. **(B)** Parasagittal CV-stained section through the cerebellum and brainstem of the cat. Scale bar 2 mm. Image from BrainMaps.org. **(C)** Parasagittal section through the monkey cerebellum at the level of the dentate nucleus. The image is from Madigan and Carpenter (1971), Figure 25, p. 40. Scale bar 1 mm. **(D)** Parasagittal section through the human cerebellum at a level lateral to the dentate nucleus. Scale bar 10 mm. The inset shows a photograph of an intact human cerebellum, inferior view, Case 188 (Witelson Normal Brain Collection, female, age 67). Scale bar in inset 1 cm.

It is obvious from a glance at pictures of the brains of different species that the human brain is distinguished by a highly expanded and intricately folded cerebral cortex (for a collection of images of the brains of humans and many other species see http://www.brainmuseum.org/index.html). It is also apparent, if less striking, from those pictures that there is a parallel expansion of the cerebellum, especially of the cerebellar hemispheres (see additional images from the studies of Voogd and Glickstein, 1998b; MacLeod et al., 2003; Sultan and Glickstein, 2007; Glickstein et al., 2009a and the inset in Figure 1D). While the basic laminar organization and cell types of the cerebellar cortex are similar across species, the surface area of cerebellar cortex increases dramatically (illustrations in Sultan and Braitenberg, 1993) and the folding pattern becomes much more intricate. Figure 1 illustrates the folding pattern of cerebellar cortex in four different species, rat (Figure 1A), cat (Figure 1B), macaque monkey (Figure 1C), and human (Figure 1D). The intricate folding pattern of the human cerebellar cortex is seen all over the cerebellum, in “old” (for example the vermis) as well as in “new” (for example the cerebellar hemispheres) regions of the cerebellum (macaque monkey cerebellar cortex is well-illustrated in Angevine, 1961).

The output of the cerebellar cortex is via the projections of the Purkinje cells to the deep cerebellar nuclei (reviews by Voogd and Glickstein, 1998a; Glickstein et al., 2009a). Along with the expansion of the cerebellar hemispheres, there is an increase in size and complexity of their target, the dentate nucleus, again uniquely so in humans (compare images of monkey and human dentate in Angevine, 1961; Madigan and Carpenter, 1971). The expansion of the cerebral cortex and the cerebellum are functionally linked: the cerebellum and the cerebral cortex are highly interconnected. As shown in many studies, the cerebellum receives topographically organized input from many functional regions of the cerebral cortex via relays in the pontine nuclei (Kuypers and Lawrence, 1967; Brodal, 1968a,b, 1971a,b, 1978; Gibson et al., 1978; Glickstein et al., 1980, 1985, 1990, 1994; Cohen et al., 1981; Baker et al., 1983; Bjaalie and Brodal, 1983; Thangnipon et al., 1983; Leergaard et al., 2006; Suzuki et al., 2012). Cortical input can also influence other pre-cerebellar nuclei (references and discussion in Kuypers and Lawrence, 1967; Suzuki et al., 2012).

The deep cerebellar nuclei in turn influence the cerebral cortex via projections to thalamic and brainstem relay sites (Orioli and Strick, 1989; Middleton and Strick, 1994, 1996, 1997a, 1998, 2000, 2001; Hoover and Strick, 1999; Dum and Strick, 2003; Kelly and Strick, 2003; Akkal et al., 2007). In addition to its contribution to cortical function, the cerebellum also can influence motor control by projections to brainstem structures like the vestibular nuclei that in turn affect movement (Langer et al., 1985).

The brainstem and thalamus, then, have a critical role in mediating cerebellar input and output. We have asked if there are parallel differences in human brainstem structures that interconnect the cerebellum and the cerebral cortex. Our analysis has shown unique features of the human brainstem that distinguish it from the brainstems of other species, including our nearest extant relative, the chimpanzee. We have also shown unique features of one component of the cerebellum, the human dentate nucleus.

How is the human brainstem unique? We have identified five principles distinguishing human brainstem organization from that of other species.

### Principle 1

There are structures that are conserved across species, but which show subtle differences in neurochemical organization.

### Principle 2

There are structures that are conserved across species, but which show *major* differences in overall organization.

### Principle 3

There are brainstem structures found in humans and chimpanzees that are not present in macaque monkeys or cats.

### Principle 4

There are brainstem structures that are found *only* in humans.

### Principle 5

Asymmetry and individual variability. There are two other unique features of the human brainstem that are usually considered exclusive characteristics of the cerebral cortex. First, there is individual variability in the size and shape of a single structure. Second, for some structures, there are left–right asymmetries in structure.

In this review, we will summarize and illustrate the evidence from published studies that led to the formulation of these five principles.

## Materials and Methods

For the figures in this review, we have photographed slides of human and chimpanzee brainstems sectioned and stained in this laboratory. We also photographed sections from the cat, macaque, squirrel monkey, and rat that had been prepared in this laboratory in the context of several different projects (Baizer and Baker, 2005, 2006b; Baizer et al., 2007, 2011a,b, 2013b,c; Baizer, 2008, 2009; Baizer and Broussard, 2010; Sultan et al., 2010). The methods and results for each project were described in detail in the individual publications. We also referred to published and online atlases of several species (BrainMaps.org and Olszewski and Baxter, 1954; Angevine, 1961; Emmers and Akert, 1963; Berman, 1968; Madigan and Carpenter, 1971; Paxinos and Huang, 1995; Franklin and Paxinos, 1997; Paxinos, 1999; Paxinos et al., 1999, 2000).

### Human tissue

Briefly, we used brainstems and cerebella from the Witelson Normal Brain Collection (Witelson and McCulloch, 1991). Use of this tissue was approved by the IRB at the University at Buffalo. For one project, the study of the inferior olive (IO), we also used samples from the Mount Sinai School of Medicine (MSSM) Brain Bank; sections from a case at the Barrow Neurological Institute were used to develop a 3D model of the principal nucleus of the inferior olive (IOpr; Baizer et al., 2011b). Table 1 shows critical parameters for the cases. For each case from the Witelson collection, the brainstem and cerebellum had been dissected away from the rest of the brain. We further dissected the cerebellum from the brainstem. Blocks of tissue were cryoprotected in 15% then 30% sucrose in 10% formalin. A small slit was made lengthwise through the pyramidal tract on one side of the brainstem to allow us to distinguish the left and right sides (this slit may be seen in Figures 5C and 11F). Frozen sections were cut in a plane transverse to the brainstem, the plane used in the atlas of Olszewski and Baxter (1954). For the MSSM cases, only a small block of tissue containing part of the IOpr was available. Those blocks were also sectioned in the transverse plane. All sections were collected and stored in plastic compartment boxes in 5% formalin. In order to have sections small enough to fit on a standard slide, we divided the cerebella into two blocks along the midsagittal plane. Some cases were embedded in albumin–gelatin. The half cerebella were sectioned in the parasagittal or coronal planes, using the atlas of Angevine (1961) to aid in the proper orientation of the blocks.

**Table 1:** **Cases and tissue history**.

Case	Source	Age	Gender	PMI	Cause of death	Year of death
155	W	50	W	9	Breast cancer	1988
158	W	51	M	1	Colorectal cancer	1989
164	W	45	W	3	Breast cancer	1991
168	W	69	M	3	Rectal cancer	1992
169	W	70	M	2	Colorectal cancer	1992
176	W	71	W	3	Colon cancer	1994
180	W	54	M	2	Adenocarcinoma	1995
188	W	67	W	2	Breast cancer	1997
1342	MSSM	36	M	17	Anaphylaxis	2006
1130	MSSM	38	M	24	Accident	2004
1057	MSSM	40	W	4	Aortic dissection	2003
1319	MSSM	48	M	17	Aortic stenosis	2006

*W, Witelson normal brain bank; MSSM, Mount Sinai School of Medicine Brain Bank*.

### Histology, immunohistochemistry

For the brainstem, we first mounted sets of sections 2 mm apart on gelled slides and stained them with CV (cresyl violet, method of LaBossiere and Glickstein, 1976). We identified the level of our sections compared to the plates in the atlas (Olszewski and Baxter, 1954). For each study, we then defined the location and rostro-caudal extent of the structure of interest, if necessary mounting and staining additional sections to define boundaries more precisely. We then used immunohistochemical techniques to examine the neurochemical properties of neurons and axons. We started with a set of antibodies that had been useful in distinguishing subdivisions of the vestibular brainstem of cats and monkeys (Baizer and Baker, 2005, 2006a,b). We subsequently added several additional markers, for example, an antibody to non-phosphorylated neurofilament protein (Baizer, 2009). We used two different visualization protocols, one using DAB-H_2_O_2_ which yields brown immunolabel and a glucose oxidase modification of that protocol (protocol described in Van Der Gucht et al., 2006) that gives gray–black immunolabel. Table 2 shows a summary of the antibodies used in all of the projects. To investigate further the suggestion of loss of IOpr neurons in the normal adult, we used a commercial silver staining kit (FD NeuroSilver Kit, FD Neurotechnologies, Columbia, MD, USA) that labels both degenerating neurons and degenerating fibers.

**Table 2:** **Primary antibodies**.

Antigen	Immunogen	Manufacturer, species, type, catalog number	Dilution
Calbindin D-28	Recombinant calbindin	Chemicon, rabbit polyclonal, #AB1778	1:1000–1:2000
Calretinin	Recombinant rat calretinin	Chemicon, rabbit polyclonal, #AB5054	1:2000–1:3000
Calretinin	Guinea pig calretinin	Chemicon, goat polyclonal, #AB1550	1:250
GAD 65/67	Synthetic peptide sequence D-F-L-I-E-E-I-E-R-L-G-Q-D-L from the C terminus of GAD	Serotec, rabbit polyclonal #AHP360	1:200
nNOS	Amino acids 1422-1433, human nNOS	Cayman, rabbit polyclonal, #160870	1:200
Neurofilament H non-phosphorylated (NPNFP)	Non-phosphorylated mammalian neurofilament H	Sternberger monoclonals/covance	1:1000–1:3000
Parvalbumin	Purified frog muscle parvalbumin	Sigma, mouse monoclonal, #P-3171	1:2000
“8B3”	Brain homogenate	Gift of Aurea Pimenta; mouse monoclonal	1:500–1:4000

### Chimpanzee brains

The analysis of the human brainstem showed enough differences from the commonly studied animals (rat, cat, macaque monkey) that we were curious if the human brain was unique or if some of these features might also be present in the brains of other great apes. We therefore extended the analysis to the chimpanzee, using three chimpanzee brains (Case AN, age 45, F; Case MT, age 25, M; Case WM, age 25, M). These brains originated from the Yerkes Primate Center and had been obtained by Drs. Hof and Sherwood (Baizer et al., 2013a,b). The brains had been immersion-fixed in 4% paraformaldehyde and subsequently stored in phosphate-buffered saline (PBS) containing 0.1% sodium azide. We sectioned blocks of tissue that included both the brainstem and the cerebellum from these three chimpanzees. Blocks were sectioned in the coronal plane to attempt to match the levels of sections to the atlases of the macaque monkey (Paxinos et al., 2000). The methods for immunohistochemistry and histology were as described for the human tissue and described in detail in Baizer et al. (2013a). We also examined archival CV-stained sections of the chimpanzee brainstem from five additional cases that had been prepared in the laboratories of Dr. Chet Sherwood and Dr. Patrick Hof.

### Data analysis

The sections were examined with a Leitz Dialux 20 light microscope, and digital images were captured with a SPOT Insight Color Mosaic camera (1200 × 1600 pixels; Diagnostic Imaging, Sterling Heights, MI, USA). Brightness and contrast of images were adjusted and plates were assembled using Adobe Photoshop CS (Adobe, San Jose, CA, USA). We used MDplot software (AccuStage, Shoreview, MN, USA) to plot the distributions of silver grain-labeled cells in the IOpr (Baizer et al., 2013c).

## Results

We have found several quantitative and qualitative differences in the organization of the brainstem between humans and other species, supporting a unique organization of the human brainstem. Unique features of the human brainstem range from relatively subtle neurochemical differences in conserved nuclei to the emergence of altogether new structures. We will summarize our data showing examples of these features in the context of the five principles outlined in the Section “Introduction.”

### Principle 1

Conserved structures that show subtle differences in neurochemical organization.

#### The neurochemical organization of the vestibular nuclear complex and related nuclei

##### A calretinin “area” in the medial vestibular nucleus

Both anatomical and physiological data on the VNC suggest that there might be functional subdivisions within the four nuclei (review and references in Baizer and Baker, 2005, 2006a,b). We examined the structure of the VNC using immunohistochemistry for calcium-binding proteins and other markers in several species. Our initial experiments, first in cats and then in monkeys were derived from experiments in the somatosensory and auditory systems showing that immunohistochemistry for calcium-binding proteins could define subdivisions within cytoarchitectonically defined structures (Rausell et al., 1992a,b; Jones et al., 1995; Molinari et al., 1995). We found that immunoreactivity to the calcium-binding proteins (CR) and calbindin (CB) showed compartments within the cytoarchitectonically defined medial vestibular nucleus (MVe).

**Figure 2:**
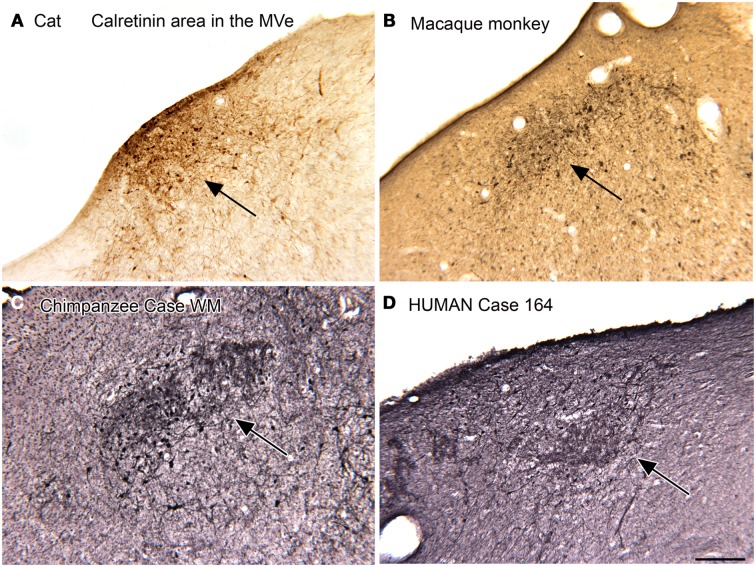
**The calretinin area of the medial vestibular nucleus in four species**. In each panel, the arrow indicates the region of calretinin-immunoreactive cells and processes. **(A)** Cat. **(B)** Macaque monkey. **(A,B)** Immunoreactivity was visualized by the DAB method. **(C)** Chimpanzee. **(D)** Human. Case 164, female, age 45. For the sections shown in **(C,D)** immunoreactivity was visualized with the glucose oxidase method. Scale bar 250 μm. MVe, medial vestibular nucleus.

We first described a small region in the MVe of the cat that had cells and processes highly immunoreactive for CR (Baizer and Baker, 2006a). We referred to this region as the “calretinin (CR) area.” We subsequently found a CR area in the MVe of other species, including squirrel and macaque monkeys, chimpanzees, and humans (Baizer and Baker, 2006a; Baizer and Broussard, 2010; Baizer et al., 2013a). Figure 2 illustrates the CR area in the cat (Figure 2A), macaque monkey (Figure 2B), chimpanzee (Figure 2C), and human (Figure 2D). While this region was present in all species, there were subtle differences among species. Specifically, there was variability in the rostro-caudal extent of the CR area relative to the extent of the parent nucleus, the MVe. In cat, the CR area was found over the total rostro-caudal extent of the MVe. In macaque monkey, the CR area extended over about 40% of the MVe and in the human only over about 20% (Baizer and Baker, 2005, 2006a; Baizer and Broussard, 2010). In all species studied, we also found a small region medial to the CR region in the MVe distinguished by intense CB immunoreactivity in fibers (Baizer and Baker, 2005, 2006a; Baizer and Broussard, 2010; Baizer et al., 2013a).

##### Neurochemical properties of the nucleus prepositus hypoglossi

**Figure 3:**
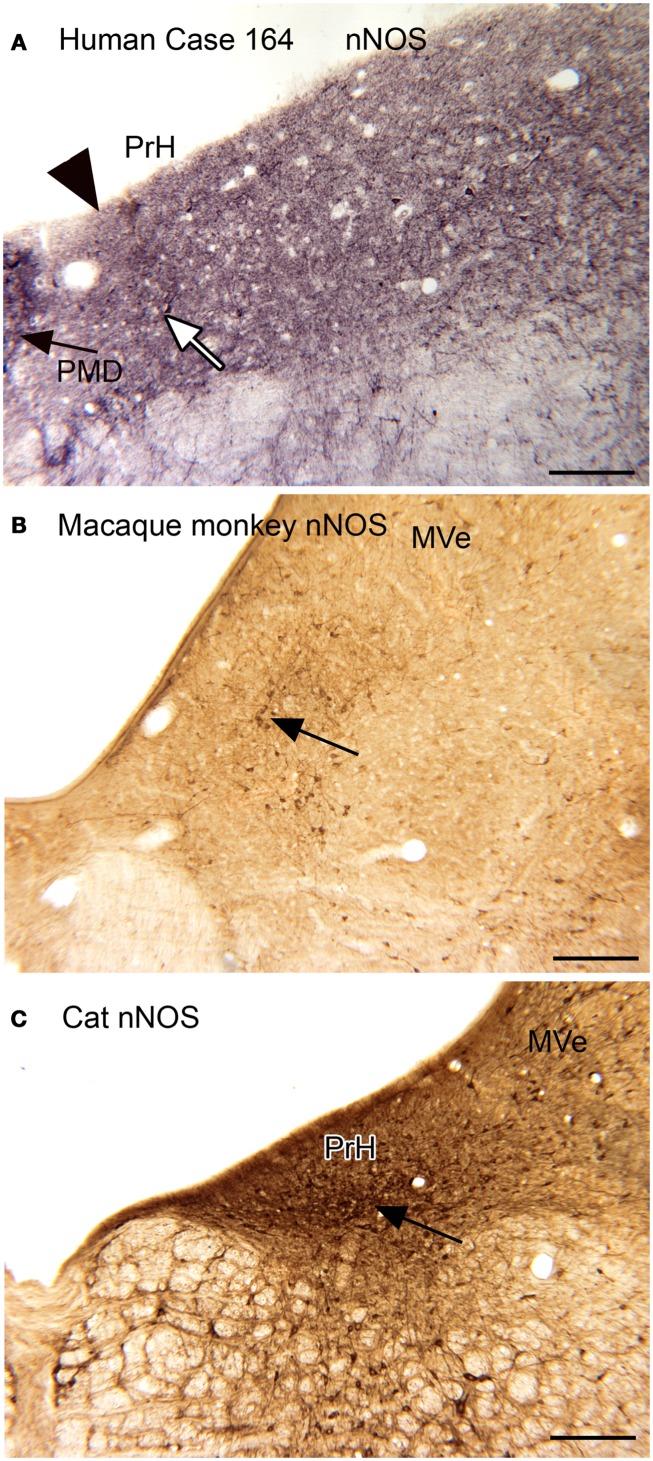
**(A)** nNOS-ir in the nucleus prepositus hypoglossi (PrH) in the human, Case 164, female, age 45. There is diffuse punctate label and a few stained neurons. The white arrow indicates one of a few stained cells. The arrowhead marks the medial border of PrH. The black arrow shows the lateral edge of the nucleus paramedianus dorsalis. Glucose oxidase visualization. Scale bar 250 μm. **(B)** nNOS-ir in PrH of macaque monkey; the arrow shows a cluster of labeled cells. Immunoreactivity visualized with DAB-H_2_O_2_. Scale bar 250 μm. **(C)** nNOS-ir in PrH of the cat. The arrow shows the ventral cluster of labeled cells. Immunoreactivity visualized with DAB-H_2_O_2_. MVe, medial vestibular nucleus.

The nucleus prepositus hypoglossi (PrH) is located in the dorsal medulla, medial to the MVe. In the cat, squirrel monkey, and macaque monkey, we found that there were neurons distinguished by the expression of nitric oxide synthase (nNOS) that were arranged in a rostro-caudal column through the nucleus (Baizer and Baker, 2006b). A subset of these neurons were also labeled by an antibody called “8B3” that had been shown to label limited cell populations in the monkey (the protein and labeling pattern are described in Pimenta et al., 2001; Dum et al., 2002). In humans and chimpanzees, however, the neurochemical organization of PrH was quite different, with only scattered nNOS-ir neurons present. We were not able to get immunostaining with the 8B3 antibody in the human sections; the target protein may not be expressed in the human. Figure 3 shows these species differences in nNOS immunoreactivity in PrH. Immunoreactivity in the cat shows many labeled cells in a ventromedial cluster of neurons (arrow in Figure 3C); in macaque monkey there is also a region of labeled cells (Figure 3B, examples at arrow) whereas in human there is only a scattering of labeled cells (example at arrow in Figure 3A).

### Principle 2 and Principle 5

Conserved structures that show *major* differences in overall organization and asymmetry and individual variability in the brainstem.

#### The principal nucleus of the inferior olive and the dentate nucleus of the cerebellum

**Figure 4:**
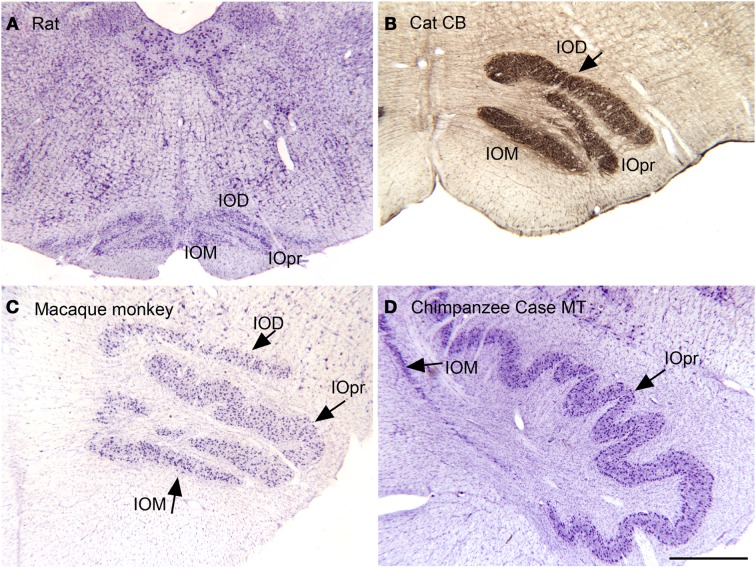
**The inferior olive (IO) in the rat [(A), CV], cat [(B), immunoreactivity to calbindin, CB], macaque monkey [(C), CV] and chimpanzee [(D), CV]**. IOD, dorsal nucleus of the inferior olive; IOM, medial nucleus of the inferior olive; IOpr, principal nucleus of the inferior olive. All images are at the same magnification. Scale bar 1 mm.

The IOpr is a structure that is conserved across species, but has major differences in organization. Figure 4 illustrates the shape of the IO in coronal sections from four species, rat (Figure 4A), cat (Figure 4B, immunostaining for calbindin), macaque monkey (Figure 4C), and chimpanzee (Figure 4D). In the chimpanzee, the shape of the IOpr has changed considerably; it has the form of a long ribbon with many infoldings.

**Figure 5:**
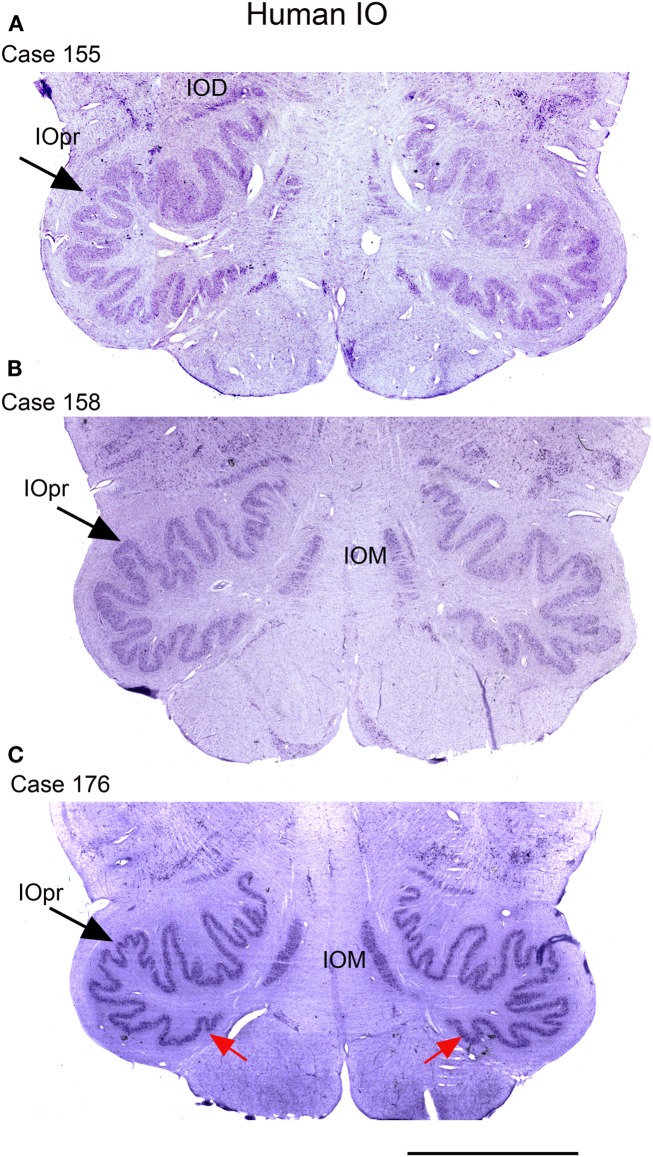
**The IO on CV sections of three different human cases**. **(A)** Case 155, female, age 50. **(B)** Case 158, male, age 51. **(C)** Case 176, female, age 71. Note differences among cases in the size and shape of the principal nucleus of the inferior olive, IOpr (compare the IOpr shape at the large arrows). Also compare the left–right folding pattern; examples of different folding shown at the red arrows. IOD, dorsal nucleus of the inferior olive; IOM, medial nucleus of the inferior olive; IOpr, principal nucleus of the inferior olive. Scale bar 5 mm.

**Figure 6:**
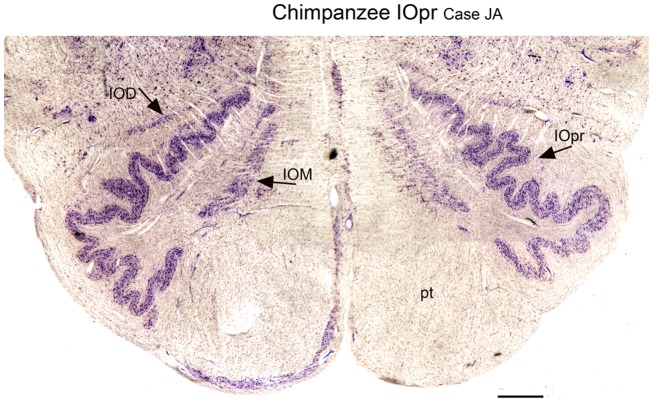
**Differences in the shape of the principal nucleus of the inferior olive on the left and right in the chimpanzee (CV)**. IOD, dorsal nucleus of the inferior olive; IOM, medial nucleus of the inferior olive; IOpr, principal nucleus of the inferior olive; pt, pyramidal tract. Scale bar 1 mm.

We next examined the size and shape of the IOpr in the human. Examination of CV-stained sections showed differences among human cases in the size, shape, and folding pattern of the IOpr. Figure 5 shows CV-stained sections through the IOpr in three different cases. While in each case the IOpr has the form of a narrow, highly folded ribbon; the configuration of the infoldings is different for each case we studied. Figure 5 also shows differences in the shape and pattern of infoldings of the IOpr on the left and right sides. We asked if these left–right differences in the IOpr might be related to handedness. We compared the volume and a measure of folding complexity of the IOpr for the left and right sides but did not find a significant difference (Baizer et al., 2011b). Left–right differences in folding pattern can also be observed in the IOpr of the chimpanzee (Figure 6).

#### IOpr degeneration in the neurologically normal adult

**Figure 7:**
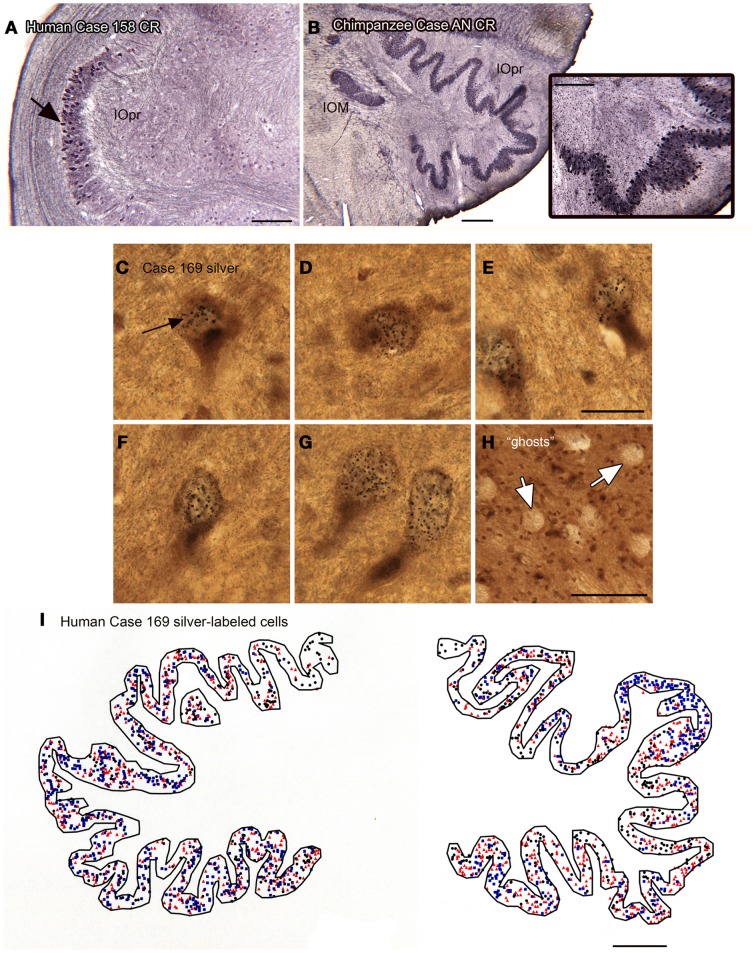
**Human–chimpanzee differences in evidence of degeneration of neurons in the IOpr**. **(A)** The arrow indicates a patch of cells showing CR-ir the human IOpr surrounded by regions with little or no CR expression. Case 158, male, age 51. Scale bar 250 μm. **(B)** Robust CR expression in neurons of the IOpr of the chimpanzee, Case AN, 45 years old. Scale bar 500 μm. The inset shows a higher magnification image of the stained cells. Scale bar in inset 250 μm. **(C–G)** Images of neurons in the IOpr of Case 169 (male, age 70) labeled with black silver grains. Scale bar 20 μm. **(H)** The arrows indicate “ghosts,” spaces the size and shape of IOpr neurons. Scale bar 100 μm. **(I)** Plot of the distribution of cells marked with silver grains on a section from Case 169. Three different symbols are used to indicate relative numbers of silver grains: black circle, few; red triangle, intermediate; blue square, many. Scale bar 1 mm.

We also found individual differences in neurochemical properties of IOpr cells. When we looked at immunostained sections, we found that some neurons in the human IOpr were immunoreactive for the calcium-binding proteins CB and CR, and also for NPNFP. However, not every neuron in the IOpr was stained for a particular marker. Figure 7A illustrates a section from one human case in which there is a dense patch of neurons immunoreactive for CR (Figure 7A, arrow), with other neurons in the structure lightly or not at all stained. The pattern of staining again varied among sections from any one case and also among cases. In many immunostained sections, there were what appeared to be “ghosts” or missing cells. These data suggested an active degenerative process with loss of IOpr neurons. We subsequently found more direct evidence of degeneration, using a silver-stain protocol to stain degenerating neurons (Baizer et al., 2013c). Figures 7C–G shows examples of neurons in the IOpr that are marked with silver grains. Figure 7H illustrates the appearance of “ghosts,” empty spaces the size and shape of IOpr neurons. Figure 7I shows a plot of the distribution of silver-labeled neurons on one section from the IOpr from one case. We asked if degeneration of neurons of the IOpr would also be seen in the chimpanzee. Figure 7B shows an image of CR-immunoreactivity in the IOpr of a chimpanzee. All neurons are darkly stained; there are no patches of more darkly stained neurons as in human (Figure 7A), this is confirmed in the inset that shows a higher magnification image of the IOpr.

#### Dentate nucleus

**Figure 8:**
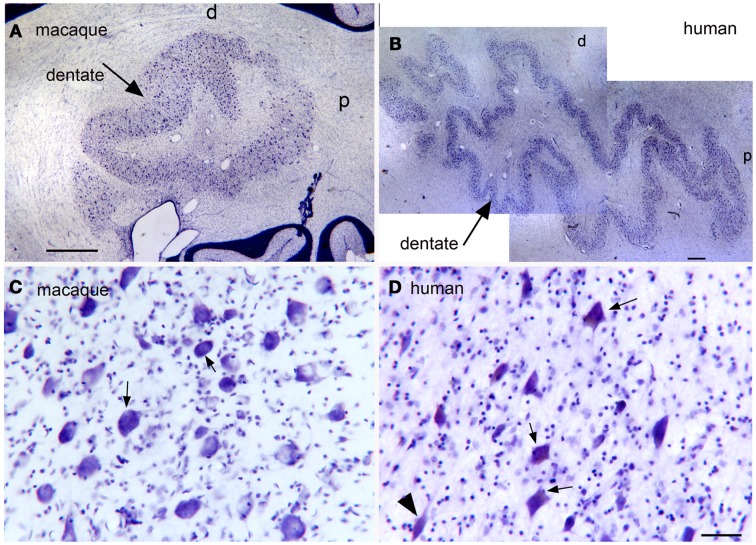
**(A)** The dentate nucleus on a CV-stained parasagittal section of the macaque monkey cerebellum. Scale bar 1 mm. **(B)** The dentate nucleus on a parasagittal CV section in the in the human cerebellum. Scale bar 1 mm. **(C)** Neurons in the dentate nucleus of the macaque are round or oval. Scale bar 50 μm. **(D)** There are many shield-shaped, distinctly multipolar, neurons in the dentate nucleus of the human. Scale bar 50 μm.

Expansion and changes in organization across primate species (Baizer, 2008; Glickstein et al., 2009b; Sultan et al., 2010). This nucleus shows both modifications in configuration and an overall expansion that are strikingly similar to the findings in the IOpr. Figures 8A,B show the dentate nucleus in parasagittal sections from a macaque monkey and a human. In the macaque (Figure 8A), the dentate appears as a closed, thick-walled roughly oval shape with only a suggestion of infoldings. In the human, however, (Figure 8B) the dentate nucleus appears as a relatively thin ribbon of constant width with many infoldings. There are also differences in the shape of the constituent large neurons. Figure 8C shows neurons in the macaque dentate have with oval cell bodies whereas the large neurons in the human dentate (Figure 8D) are polygonal or shield-shaped.

**Figure 9:**
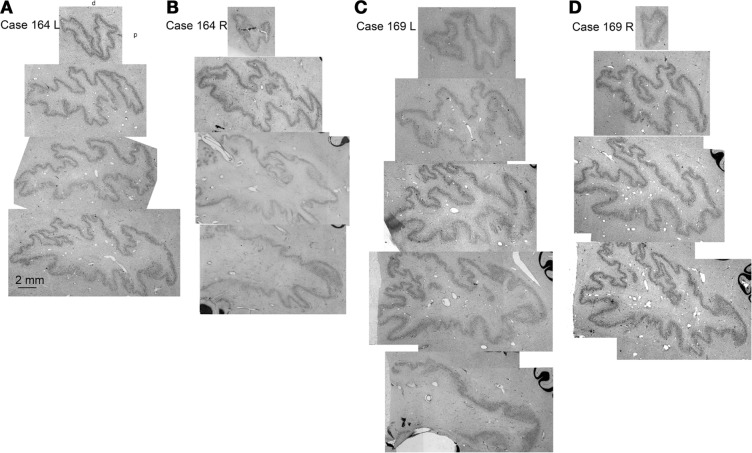
**Individual variability in the shape and folding pattern of the dentate nucleus**. **(A–D)** The dentate nucleus on parasagittal sections about 2 mm apart. **(A,B)** Show the left and right sides of Case 164 (female, age 45) and **(C,D)** show the left and right sides of Case 169 (male, age 70). The images are scans of CV-stained sections from a flat bed scanner.

Since the two halves of the cerebellum were cut separately, we cannot show left–right differences in folding pattern on a single section, as we could for the IOpr. However, comparing sections within and among cases (Figure 9) again suggest both individual variability in the pattern of infoldings of the dentate ribbon and left–right differences in the folding pattern within a single case.

### Principle 3

Structures found only in apes and humans.

#### The nucleus paramedianus dorsalis

**Figure 10:**
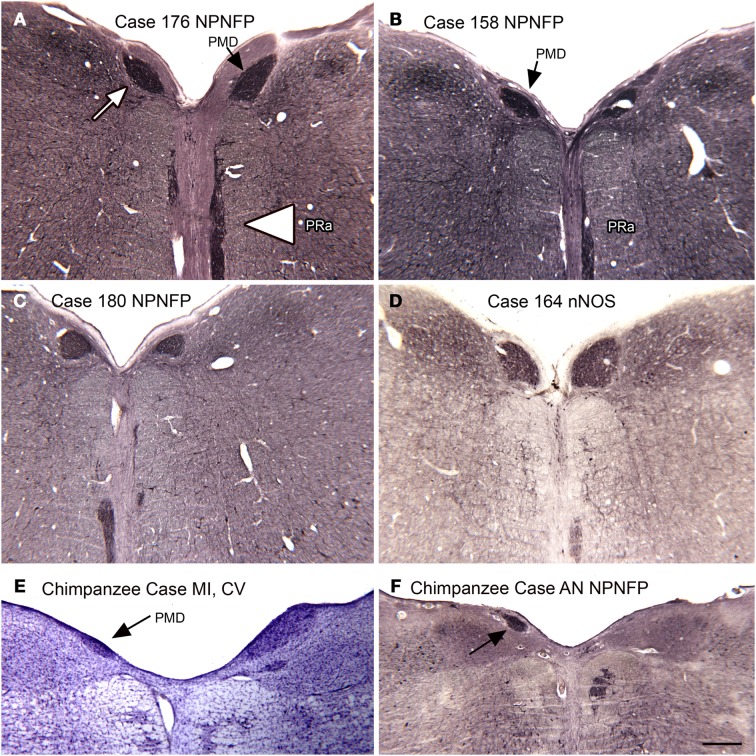
**The nucleus paramedianus (PMD) and the nucleus pararaphales (PRa) in the human and chimpanzee**. **(A)** The arrow shows the darkly stained PMD, the arrowhead the PRa. Case 176, female, age 71. **(B)** Case 158, male, age 51. **(C)** Case 180, male, age 54. **(D)** Case 164, female, age 45. **(E,F)** PMD (arrows) in the two chimpanzees. Scale bar 500 μm. Abbreviations, nNOS, nitric oxide synthase; NPNFP, non-phosphorylated neurofilament protein.

In the course of examining the vestibular nuclear complex in the human, we noticed a small, medially located nucleus that we had not seen in either the cat or the monkey (Baizer et al., 2007). Moreover, this nucleus is not illustrated in the atlases of these species (Berman, 1968; Paxinos et al., 2000). We did, however, find this nucleus in the Olszewski and Baxter (1954) atlas of the human brainstem. That atlas called it the nucleus PMD. Figures 10A–D illustrate PMD (arrow in Figure 10A) in four different human cases; there are noteworthy differences in size and shape among cases. Neurons in the PMD express NPNFP (Figures 10A–C) and nNOS (Figure 10D). This nucleus is also present in the brainstem of the chimpanzee (Figures 10E,F), in agreement with Brodal (1983).

### Principle 4

There are brainstem structures that are found *only* in humans and Principle 5. Left–right asymmetry and individual variability.

#### The nucleus pararaphales and the arcuate nucleus (Baizer and Broussard, 2010; Baizer et al., 2013b)

##### Pararaphales

Many studies have shown that the antibody to non-phosphorylated neurofilament protein, NPNFP (the antibody is often referred to by its catalog number, “SMI32”) to be a very useful marker for distinguishing cortical structure (Hof and Morrison, 1995; Van Der Gucht et al., 2006). This antibody is also useful in distinguishing cell populations in the brainstem (Baizer, 2009; Baizer et al., 2011a). In NPNFP-ir sections of the human brainstem, we saw a distinctive pattern of staining along the midline. The stained region best corresponded to a nucleus called the nucleus pararaphales in the atlas of Olszewski and Baxter (1954). We then re-examined NPNFP-ir sections of cats and monkeys and did not find a similar staining pattern. Figure 10A (arrowhead) shows this nucleus in the human; comparison of the sections from different cases (Figures 10A–D) reveals both variability in size of this nucleus among cases and left–right asymmetry within a single case.

##### Arcuate nucleus

**Figure 11:**
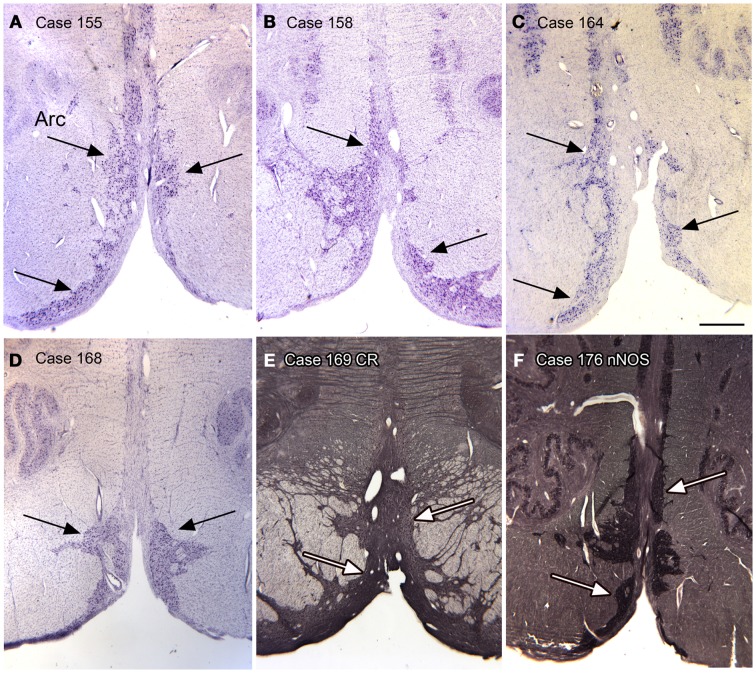
**The arcuate nucleus (Arc, arrows) in six human cases; (A–D) CV; (E) Calretinin**. **(F)** nNOS. Scale bar 1 mm.

In looking at transverse sections of the humans’ brainstem in rostral medulla and continuing rostral to the level of the pontine nuclei, one is struck by the presence of a large bilateral cell group, termed the arcuate nuclei on the edge of the pyramidal tract (Olszewski and Baxter, 1954). Figures 11A–F shows the presence of this cell group in six cases. We found this cell group to be a reliable feature of the brainstem of every human case that we examined. We also found that the cells in the Arc expressed CR and nNOS (Figures 11E,F). Although we did not do any quantitative analysis of left–right differences in this structure, qualitative differences are apparent.

**Figure 12:**
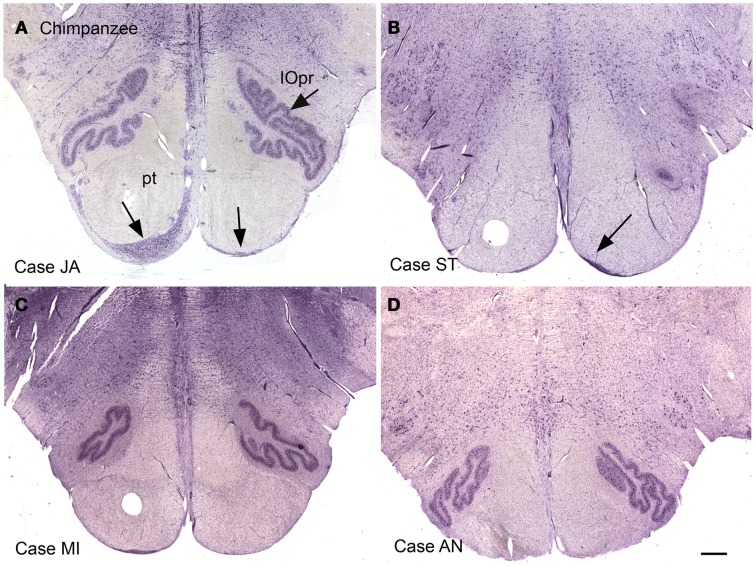
**The ventral medulla in the chimpanzee, Cases JA (A), ST (B), MI (C), and AN (D)**. **(A)** The arrows show stained cells ventral to the pyramidal tracts in the expected location of the arcuate nucleus. **(B)** The arrow shows a small group of stained cells. **(C,D)** There are no stained neurons ventral to the pyramidal tracts in Case AN or Case MI. Scale bar 1 mm.

Again, this nucleus was not seen in our examination of the brainstems of cat, squirrel monkey, or macaque monkey, nor is it shown in the standard brain atlases for these species (Berman, 1968; Paxinos et al., 2000). We then looked for this nucleus in the chimpanzee brainstem and found surprising differences among the cases we examined. Figures 12A–D shows images of the ventral brainstem in four chimpanzees. In one of them (Case JA, Figure 12A) there are cells located ventral to the pyramidal tract on the left in the expected location of the arcuate nucleus. In a second case (Case ST, Figure 12B, arrow), there were a very few cells ventral to the pyramidal tract on the right. These data suggest that the arcuate nucleus is not a regular feature of the chimpanzee brainstem, but that it may be worth examining more cases or sections from other great apes to see if the cells in Case JA are a developmental anomaly unique to this animal.

## Discussion

The human brain is distinguished by parallel and functionally linked expansion of the cerebellum and the cerebral cortex (Herculano-Houzel, 2010). Our studies show that there are also major changes in the human brainstem, most notably in structures that are known or suspected to project to the cerebellum. It is clear that the expansion of the human brain underlies unique aspects of human cognitive and motor function. What is known about the relative contributions of the cerebellum and the cerebral cortex? The cerebral cortex is critical for both cognitive function and motor control. The traditional view of the cerebellum is that it is critical for motor, but not cognitive, function. That view has been challenged on the basis of anatomical, physiological, and behavioral data, with many supporting role for the cerebellum in cognitive functions (Kim et al., 1994; Middleton and Strick, 1994, 1996, 1997b, 1998, 2000; Schmahmann, 1998; Schmahmann and Sherman, 1998; Dum et al., 2002), but see Glickstein (1993, 2007) for an opposing point of view.

We will focus on the motor role of the cerebellum and associated brainstem structures. Humans are bipedal and bipedal locomotion imposes very different demands for the control of balance and posture, functions to which the cerebellum contributes (reviewed in Morton and Bastian, 2004). Second, bipedal locomotion frees the forelimbs and hands, allowing the development of fine motor skills, skilled tool use, and the emergence of handedness. There are parallel changes in the visual system, with the evolution of the fovea and parallel changes in voluntary eye movements (Glickstein, 1969; Franco et al., 2000; Horn and Leigh, 2011). The cerebellum also participates in the control of the hands and fingers as well as in the control of eye movements (Glickstein et al., 2005). The specializations of primate brainstem structures may be related to these evolutionary changes.

### MVe, PrH, PMD: Balance, posture, eye movements

We saw neurochemical differences in the MVe and PrH among species. We also found a nucleus, the PMD, present only in apes. What functional differences might these anatomical differences reflect? As a general hypothesis, the differences in MVe organization may reflect different processing of vestibular information required by bipedal locomotion. It is not yet possible to relate the differences in the extent of the CR area of the MVe to different functional demands in different species. While the presence of the CR area has been established in many species, its function has not yet been investigated directly.

We also saw neurochemical differences among species in PrH. The functions of PrH are better established than the functions of the CR area. It has a major role in the generation of saccadic eye movements in that it provides eye position signals to abducens neurons (Kaneko and Fuchs, 1991; Kaneko, 1992, 1997, 1999). There are differences in the organization of the retina between cats and monkeys, with cats having only an area centralis and monkeys having a fovea (Polyak, 1957, pp. 281–285). There are also major differences in the parameters of saccadic eye movements between cats and monkeys (Fuchs, 1967; Stryker and Blakemore, 1972), and subtle differences in saccadic eye movements in humans compared to macaque monkeys (Collewijn et al., 1988a,b; Baizer and Bender, 1989). The changes in PrH may reflect differences in the circuitry required for programing saccadic eye movements.

The nucleus PMD is found in apes and humans, but not in cats or monkeys. It is located just medial to PrH. It has not been studied by anatomical, physiological, or behavioral techniques, so there is no direct evidence about its connections or function. A plausible speculation about this region is that it is a derivative of the PrH, which has been shown to have functional subdivisions (discussion and references in Baizer et al., 2007). If that is true, further speculation would suggest that PMD is a pre-cerebellar nucleus with a role in the programing or control of eye movements. Since this nucleus is not seen in animals in which invasive experiments are possible, its function will remain mysterious for the present. It is possible that as imaging techniques increase in sensitivity a role for PMD in eye movements may be directly confirmed.

### Arcuate nucleus and PRa

The functions and connections of the arcuate nucleus remain a mystery. It was suggested by Olszewski and Baxter (1954) that the neurons of the arcuate nucleus were displaced pontine neurons, which would make them pre-cerebellar neurons. However, our neurochemical data did not support this view; neurons in the human pontine nuclei expressed the calcium-binding protein parvalbumin whereas neurons in the Arc did not (Baizer and Broussard, 2010). We therefore argued that the Arc is indeed a new nucleus. There is one report identifying an “arcuate” nucleus in the mouse, however the location and neurochemical properties of the neurons described in the mouse suggest that they are instead a component of the IO (Fu and Watson, 2012).

The functions of the PRa are even more mysterious as there are no data about its embryonic origins or connections. The expression of NPNFP in this nucleus suggests that these neurons may have long projections. Candidate targets include the spinal cord and the cerebellum.

### IOpr and dentate nucleus: Individual differences and left–right asymmetry

Individual differences in structure among cases and left–right asymmetries in a single case are usually considered exclusively cortical attributes, but were prominent and obvious for the human IOpr and the dentate nucleus. There may also be more subtle individual differences and/or asymmetries in other human or ape brainstem nuclei that would be revealed by more quantitative analysis. How do the studies of cerebral cortex color the interpretation of these findings?

Both individual differences in cortical sulcal or gyral morphology and structural asymmetry are extensively documented in humans (Galaburda et al., 1978; Steinmetz et al., 1989; Falk et al., 1991; Jancke et al., 1994; Anderson et al., 1999; Westbury et al., 1999; Witelson et al., 1999). Do these structural differences correlate with functional differences or do they reflect genetic drift or random developmental changes with no functional significance? The majority of studies attempting to correlate structure and function in the human brain have been done in the context of two human behaviors, language, and handedness. Both language and handedness are recognized as lateralized functions, with most people being right-handed and left-hemisphere dominant for language (Knecht et al., 2000). Language has been linked to cortical asymmetry, particularly of the planum temporale (Geschwind and Levitsky, 1968; Galaburda et al., 1978; Geschwind, 1978; Steinmetz, 1996). Handedness is also established as a lateralized function, but differs in that there is a range of hand preferences in the population (Annett, 1967; Amunts et al., 2000). Language lateralization and handedness are correlated; in some (percentages vary among studies) left-handed people language is controlled by the right hemisphere (Pujol et al., 1999; Knecht et al., 2000; Hund-Georgiadis et al., 2002). Handedness has been correlated with left–right asymmetry in the structure of motor cortex, although not all studies are in agreement on this point (reviews in Amunts et al., 2000; Hammond, 2002).

### Asymmetry and individual differences in the IOpr

Neither individual differences nor left–right asymmetry has been systematically analyzed for subcortical structures. We found both to be major features of the IOpr. The IO is present in all vertebrates that have a cerebellum, but it varies dramatically in size and shape among different species (Kooy, 1917, Figure 4). The IOpr is one of the most distinctive structures of the ape brainstem. In comparing sections through the medulla across species, it appears that in chimpanzees and even more dramatically in humans, the entire ventral medulla has expanded outward to contain the enlarged IOpr (see Figure 5). Do the left–right differences in the IOpr structure correlate with a functional asymmetry? Our initial hypothesis was that the structural differences in the human IOpr are driven by handedness (Our studies have so far been limited to right-handed cases from the Witelson Normal Brain collection). To test this idea, we compared two measurements for the left and right, volume and an index of folding complexity. We did not find that the IOpr was larger or had a more complex folding pattern in the side contralateral to the preferred hand (Baizer et al., 2011b). There may be more subtle measurements like cell packing density, sizes of cells, thickness of the IOpr ribbon, or total number of cells that would show differences correlated with handedness. Similarly, the dentate nucleus asymmetries could be related to motor functions like handedness and skilled movement.

The IOpr projects to the dentate nucleus both directly and via the cerebellar hemispheres (Courville et al., 1977; Brodal and Brodal, 1981; Ruigrok and Voogd, 2000). It has been suggested that the dentate nucleus has a role in cognition (Dum et al., 2002). It is therefore possible that the IOpr or dentate nucleus or both contribute to a lateralized cognitive function like language.

The other major difference between the IOpr in humans and all other species, including the chimpanzee, was the presence of apparently degenerating cells and the presence of “ghosts,” empty spaces that appeared to represent degenerated neurons. The idea that neurons in the adult IOpr may degenerate is not a new one. Olszewski and Baxter (1954) noted that “these cells (IO) tend to accumulate lipofuscin early in life and the Nissl stained sections of “normal” persons over middle age may show a striking paucity of cells.” We saw evidence of IOpr neuronal degeneration in every case examined. While we have not yet quantified the loss of IOpr cells as a function of age, our hypothesis is that this neuronal loss is an age-related process, with a broad distribution of age of onset and individual variability in the time course. (The best analogy may be graying hair as a function of age. Graying hair is clearly an age-related process, but there is great individual variability both in the age of onset and in the speed of the process.) This IOpr degeneration appears to be a normal process, in contrast to the phenomenon of olivary hypertrophy seen after lesions of afferent and efferent structures (Goto et al., 1988; Kitajima et al., 1994; Krings et al., 2003; Marden, 2013).

Our present understanding of the circuitry of cerebellar cortex suggests that the loss of IOpr neurons should have profound consequences for cerebellar function. The IO is the sole source of climbing fibers (Armstrong, 1974). The climbing fibers provide critical input that results in the generation of complex spikes in Purkinje cells (review in Eccles et al., 1966; Gibson et al., 2004). Several authors have suggested that climbing fiber input and the generation of complex spikes are critical for cerebellar motor learning (Gilbert and Thach, 1977; review and references in Morton and Bastian, 2004; Yang and Lisberger, 2013). The loss of IOpr neurons in the human should then have major functional consequences for the impairment of motor learning with age (references in Fraser et al., 2009). This loss might contribute to impairments of balance and the increase of falls in the elderly (Matheson et al., 1999; Bloem et al., 2003).

It is important to note that individual differences and functional asymmetry of cerebral cortex are not uniquely human. Various other species have shown hand preferences (Lehman, 1978; Annett and Annett, 1991; Phillips and Sherwood, 2005), although not all species show the bias toward right-handedness shown in humans (Lehman, 1978). There is also some evidence for left–right asymmetries in motor cortex. Asymmetries in cortical organization have also been reported for non-human primates for putative “language” cortex (discussion and references in Gannon et al., 1998). Individual differences in cortical morphology in other regions of macaque monkey and chimpanzee cortex have been described (Cheverud et al., 1990; Van Der Gucht et al., 2006; Cantalupo et al., 2009). However, the analysis of individual variability in brain structure and abilities is a neglected approach for much animal-based experimental neuroscience. There are atlases of the brains of the most commonly studied species (Emmers and Akert, 1963; Berman, 1968; Franklin and Paxinos, 1997; Paxinos, 1999; Paxinos et al., 1999, 2000). The assumption behind an atlas is that one mouse or rat brain is essentially the same as the next. Similarly, traditional tract-tracing and electrophysiological studies are based on a limited number of cases; the connections or response properties as studied in a few animals are assumed to hold true for the entire normal population. Clearly the possibility of individual differences needs to be considered at least in primates. It is also important to recognize, however, that not all individual differences may be functionally significant; some differences may simply reflect chance factors affecting brain development.

The disregard of individual variability also colors some studies of the human brain. For example, it is often assumed that the individual brain shown in typical human brain atlases (Olszewski and Baxter, 1954; Talairach and Tournoux, 1988; Paxinos and Huang, 1995; Mai et al., 2008) represents a model for all human brains, especially with respect to subcortical structures. Such an assumption reinforces the disregard of individual differences. The technology for imaging the human brain has developed rapidly over the last decades (Le Bihan et al., 2001; Chabert et al., 2005; Alvarez-Linera, 2008; Cole et al., 2010). Imaging is widely used in the study of human brain functions (a 2013 PubMed search for “fMRI” returns over 7000 references). However, the results of many imaging studies are shown on standard depictions of the brain, with data averaged across subjects (some examples Petersen et al., 1990; Zatorre et al., 1994; Tzourio-Mazoyer et al., 2002; Eickhoff et al., 2005). The consequence is that individual differences in brain activity or structure may be averaged out rather than studied as an interesting and important feature.

An obligatory aspect of human evolution appears to be major variability from one person to the next in both motor and cognitive skills. For example, compare the ability to maintain balance and posture of an Olympic gymnast or the fine motor skills of a surgeon or violinist with those of an “average” person. Similarly, there is a broad distribution of assessments of cognitive function like scores on IQ tests. Some people easily learn 10 languages, others struggle with one. Presumably individual differences in motor and cognitive function are reflected in subtle, as well as not so subtle, individual differences in brain structure. There is also a small, and rather controversial, literature on individual differences in brain structure related to variables like gender, sexual orientation, and cognitive function (some examples: Holloway and de Lacoste, 1986; Witelson, 1989, 1991; McCormick et al., 1990; Levay, 1991; Witelson and Goldsmith, 1991; Allen and Gorski, 1992; Witelson and Kigar, 1992; Shaywitz et al., 1995; Witelson et al., 1995, 1999). While often neglected in neuroscience, the recognition of individual differences is of increasing concern in the development of individualized medical treatments based on genetic profiles (van’t Veer et al., 2002; Chang et al., 2003; Moroni et al., 2005; Sartore-Bianchi et al., 2007).

### Unique status of cerebral cortex and the concept of the “reptilian brain”

The idea that evolution affects only the cerebral cortex, with brainstem and cerebellum essentially unchanged entered the popular culture of neuroscience through the writings of Paul Maclean, “The Triune Brain” (MacLean, 1990) and Carl Sagan’s “reptilian brain” (Sagan, 1977). The concept of the “reptilian brain” maintains that the brainstem and cerebellum are “old” structures that have not changed over evolution. That perspective still colors the understanding of students and the general public today. As shown in this review, it clearly does not reflect the dramatic changes in cerebellar and brainstem structures and their contribution to uniquely human capabilities.

## Conflict of Interest Statement

The author declares that the research was conducted in the absence of any commercial or financial relationships that could be construed as a potential conflict of interest.

## References

[B1] Akkal D. Dum R. P. Strick P. L. (2007). Supplementary motor area and presupplementary motor area: targets of basal ganglia and cerebellar output. J. Neurosci. 27, 10659–10673 10.1523/JNEUROSCI.3134-07.2007 17913900 PMC6672811

[B2] Allen L. S. Gorski R. A. (1992). Sexual orientation and the size of the anterior commissure in the human brain. Proc. Natl. Acad. Sci. U.S.A. 89, 7199–7202 10.1073/pnas.89.15.7199 1496013 PMC49673

[B3] Alvarez-Linera J. (2008). 3T MRI: advances in brain imaging. Eur. J. Radiol. 67, 415–426 10.1016/j.ejrad.2008.02.045 18455895

[B4] Amunts K. Jancke L. Mohlberg H. Steinmetz H. Zilles K. (2000). Interhemispheric asymmetry of the human motor cortex related to handedness and gender. Neuropsychologia 38, 304–312 10.1016/S0028-3932(99)00075-5 10678696

[B5] Anderson B. Southern B. D. Powers R. E. (1999). Anatomic asymmetries of the posterior superior temporal lobes: a postmortem study. Neuropsychiatry Neuropsychol. Behav. Neurol. 12, 247–254 10527109

[B6] Angevine J. B. (1961). The Human Cerebellum: An Atlas of Gross Topography in Serial Sections. Boston: Little, Brown and Company

[B7] Annett M. (1967). The binomial distribution of right, mixed and left handedness. Q. J. Exp. Psychol. 19, 327–333 10.1080/14640746708400109 6080917

[B8] Annett M. Annett J. (1991). Handedness for eating in gorillas. Cortex 27, 269–275 10.1016/S0010-9452(13)80131-1 1879155

[B9] Armstrong D. M. (1974). Functional significance of connections of the inferior olive. Physiol. Rev. 54, 358–417 4362162 10.1152/physrev.1974.54.2.358

[B10] Baizer J. S. (2008). “Individual variability in the organization of the human dentate nucleus,” in Neuroscience Meeting Planner (Washington, DC: Society for Neuroscience), Program No. 471.2.

[B11] Baizer J. S. (2009). Nonphosphorylated neurofilament protein is expressed by scattered neurons in the vestibular and precerebellar brainstem. Brain Res. 1298, 46–56 10.1016/j.brainres.2009.08.073 19728992 PMC2761759

[B12] Baizer J. Baker J. (2005). Immunoreactivity for calcium-binding proteins defines subregions of the vestibular nuclear complex of the cat. Exp. Brain Res. 164, 78–91 10.1007/s00221-004-2211-8 15662522 PMC1201542

[B13] Baizer J. S. Baker J. F. (2006a). Immunoreactivity for calretinin and calbindin in the vestibular nuclear complex of the monkey. Exp. Brain Res. 172, 103–113 10.1007/s00221-005-0318-1 16369782

[B14] Baizer J. S. Baker J. F. (2006b). Neurochemically defined cell columns in the nucleus prepositus hypoglossi of the cat and monkey. Brain Res. 1094, 127–137 10.1016/j.brainres.2006.03.113 16701575

[B15] Baizer J. S. Baker J. F. Haas K. Lima R. (2007). Neurochemical organization of the nucleus paramedianus dorsalis in the human. Brain Res. 1176, 45–52 10.1016/j.brainres.2007.08.017 17869228 PMC2078602

[B16] Baizer J. S. Bender D. B. (1989). Comparison of saccadic eye movements in humans and macaques to single-step and double-step target movements. Vision Res. 29, 485–495 10.1016/0042-6989(89)90011-4 2781737

[B17] Baizer J. S. Broussard D. M. (2010). Expression of calcium-binding proteins and nNOS in the human vestibular and precerebellar brainstem. J. Comp. Neurol. 518, 872–895 10.1002/cne.22250 20058225

[B18] Baizer J. Paolone N. A. Witelson S. F. (2011a). Nonphosphorylated neurofilament protein is expressed by scattered neurons in the human vestibular brainstem. Brain Res. 1382, 45–56 10.1016/j.brainres.2011.01.079 21281611

[B19] Baizer J. S. Sherwood C. C. Hof P. R. Witelson S. F. Sultan F. (2011b). Neurochemical and structural organization of the principal nucleus of the inferior olive in the human. Anat. Rec. 294, 1198–1216 10.1002/ar.21400 21630474

[B20] Baizer J. S. Paolone N. A. Sherwood C. C. Hof P. R. (2013a). Neurochemical organization of the vestibular brainstem in the common chimpanzee (*Pan troglodytes*). Brain Struct. Funct. 218, 1463–1485 10.1007/s00429-012-0470-x 23179862

[B21] Baizer J. S. Weinstock N. Witelson S. F. Sherwood C. C. Hof P. R. (2013b). The nucleus pararaphales in the human, chimpanzee, and macaque monkey. Brain Struct. Funct. 218, 389–403 10.1007/s00429-012-0403-8 22426796

[B22] Baizer J. S. Wong K. M. Hof P. R. Witelson S. F. Sherwood C. C. (2013c). “Degeneration of neurons in the principal nucleus of the inferior olive of the human: evidence from silver staining,” in Neuroscience Meeting Planner (San Diego, CA: Society for Neuroscience), Program No. 469.416.

[B23] Baker J. Gibson A. Mower G. Robinson F. Glickstein M. (1983). Cat visual corticopontine cells project to the superior colliculus. Brain Res. 265, 227–232 10.1016/0006-8993(83)90336-0 6850325

[B24] Berman A. (1968). The Brain Stem of the Cat. Madison, WI: University of Wisconsin Press

[B25] Bjaalie J. G. Brodal P. (1983). Distribution in area 17 of neurons projecting to the pontine nuclei: a quantitative study in the cat with retrograde transport of HRP-WGA. J. Comp. Neurol. 221, 289–303 10.1002/cne.902210305 6689170

[B26] Bloem B. R. Steijns J. A. Smits-Engelsman B. C. (2003). An update on falls. Curr. Opin. Neurol. 16, 15–26 10.1097/00019052-200302000-00003 12544853

[B27] Brodal A. (1983). The perihypoglossal nuclei in the macaque monkey and the chimpanzee. J. Comp. Neurol. 218, 257–269 10.1002/cne.902180303 6886074

[B28] Brodal P. (1968a). The corticopontine projection in the cat. Demonstration of a somatotopically organized projection from the second somatosensory cortex. Arch. Ital. Biol. 106, 310–312 5760107

[B29] Brodal P. (1968b). The corticopontine projection in the cat. I. Demonstration of a somatotopically organized projection from the primary sensorimotor cortex. Exp. Brain Res. 5, 210–234 10.1007/BF00238665 5721752

[B30] Brodal P. (1971a). The corticopontine projection in the cat. I. The projection from the proreate gyrus. J. Comp. Neurol. 142, 127–139 10.1002/cne.901420202 4106737

[B31] Brodal P. (1971b). The corticopontine projection in the cat. II. The projection from the orbital gyrus. J. Comp. Neurol. 142, 141–151 10.1002/cne.901420203 4106738

[B32] Brodal P. (1978). The corticopontine projection in the rhesus monkey. Origin and principles of organization. Brain 101, 251–283 10.1093/brain/101.2.251 96910

[B33] Brodal P. Brodal A. (1981). The olivocerebellar projection in the monkey. Experimental studies with the method of retrograde tracing of horseradish peroxidase. J. Comp. Neurol. 201, 375–393 10.1002/cne.902010306 7276256

[B34] Cantalupo C. Oliver J. Smith J. Nir T. Taglialatela J. P. Hopkins W. D. (2009). The chimpanzee brain shows human-like perisylvian asymmetries in white matter. Eur. J. Neurosci. 30, 431–438 10.1111/j.1460-9568.2009.06830.x 19614754 PMC4195238

[B35] Chabert S. Molko N. Cointepas Y. Le Roux P. Le Bihan D. (2005). Diffusion tensor imaging of the human optic nerve using a non-CPMG fast spin echo sequence. J. Magn. Reson. Imaging 22, 307–310 10.1002/jmri.20383 16028249

[B36] Chang J. C. Wooten E. C. Tsimelzon A. Hilsenbeck S. G. Gutierrez M. C. Elledge R. (2003). Gene expression profiling for the prediction of therapeutic response to docetaxel in patients with breast cancer. Lancet 362, 362–369 10.1016/S0140-6736(03)14023-8 12907009

[B37] Cheverud J. M. Falk D. Vannier M. Konigsberg L. Helmkamp R. C. Hildebolt C. (1990). Heritability of brain size and surface features in rhesus macaques (*Macaca mulatta*). J. Hered. 81, 51–57 2332614 10.1093/oxfordjournals.jhered.a110924

[B38] Cohen J. L. Robinson F. May J. Glickstein M. (1981). Corticopontine projections of the lateral suprasylvian cortex: de-emphasis of the central visual field. Brain Res. 219, 239–248 10.1016/0006-8993(81)90289-4 7260631

[B39] Cole D. M. Smith S. M. Beckmann C. F. (2010). Advances and pitfalls in the analysis and interpretation of resting-state FMRI data. Front. Syst. Neurosci. 4:8 10.3389/fnsys.2010.00008 20407579 PMC2854531

[B40] Collewijn H. Erkelens C. J. Steinman R. M. (1988a). Binocular co-ordination of human horizontal saccadic eye movements. J. Physiol. 404, 157–182 3253429 10.1113/jphysiol.1988.sp017284 PMC1190820

[B41] Collewijn H. Erkelens C. J. Steinman R. M. (1988b). Binocular co-ordination of human vertical saccadic eye movements. J. Physiol. 404, 183–197 3253430 10.1113/jphysiol.1988.sp017285 PMC1190821

[B42] Courville J. Augustine J. R. Martel P. (1977). Projections from the inferior olive to the cerebellar nuclei in the cat demonstrated by retrograde transport of horseradish peroxidase. Brain Res. 130, 405–419 10.1016/0006-8993(77)90105-6 890443

[B43] Dum R. P. Li C. Strick P. L. (2002). Motor and nonmotor domains in the monkey dentate. Ann. N. Y. Acad. Sci. 978, 289–301 10.1111/j.1749-6632.2002.tb07575.x 12582061

[B44] Dum R. P. Strick P. L. (2003). An unfolded map of the cerebellar dentate nucleus and its projections to the cerebral cortex. J. Neurophysiol. 89, 634–639 10.1152/jn.00626.2002 12522208

[B45] Eccles J. C. Llinas R. Sasaki K. (1966). The excitatory synaptic action of climbing fibres on the Purkinje cells of the cerebellum. J. Physiol. 182, 268–296 5944665 10.1113/jphysiol.1966.sp007824 PMC1357472

[B46] Eickhoff S. B. Stephan K. E. Mohlberg H. Grefkes C. Fink G. R. Amunts K. (2005). A new SPM toolbox for combining probabilistic cytoarchitectonic maps and functional imaging data. Neuroimage 25, 1325–1335 10.1016/j.neuroimage.2004.12.034 15850749

[B47] Emmers R. Akert K. (1963). A Stereotaxic Atlas of the Brain of the Squirrel Monkey (Saimiri sciureus). Madison: University of Wisconsin Press

[B48] Falk D. Hildebolt C. Cheverud J. Kohn L. A. Figiel G. Vannier M. (1991). Human cortical asymmetries determined with 3D MR technology. J. Neurosci. Methods 39, 185–191 10.1016/0165-0270(91)90084-D 1798346

[B49] Franco E. C. Finlay B. L. Silveira L. C. Yamada E. S. Crowley J. C. (2000). Conservation of absolute foveal area in new world monkeys. A constraint on eye size and conformation. Brain Behav. Evol. 56, 276–286 10.1159/000047211 11251320

[B50] Franklin K. B. J. Paxinos G. (1997). The Mouse Brain in Stereotaxic Coordinates. San Diego: Academic Press

[B51] Fraser S. A. Li K. Z. Penhune V. B. (2009). A comparison of motor skill learning and retention in younger and older adults. Exp. Brain Res. 195, 419–427 10.1007/s00221-009-1806-5 19404628

[B52] Fu Y. H. Watson C. (2012). The arcuate nucleus of the C57BL/6J mouse hindbrain is a displaced part of the inferior olive. Brain Behav. Evol. 79, 191–204 10.1159/000335032 22301572

[B53] Fuchs A. F. (1967). Saccadic and smooth pursuit eye movements in the monkey. J. Physiol. 191, 609–631 4963872 10.1113/jphysiol.1967.sp008271 PMC1365495

[B54] Galaburda A. M. Sanides F. Geschwind N. (1978). Human brain. Cytoarchitectonic left-right asymmetries in the temporal speech region. Arch. Neurol. 35, 812–817 10.1001/archneur.1978.00500360036007 718483

[B55] Gannon P. J. Holloway R. L. Broadfield D. C. Braun A. R. (1998). Asymmetry of chimpanzee planum temporale: humanlike pattern of Wernicke’s brain language area homolog. Science 279, 220–222 10.1126/science.279.5348.220 9422693

[B56] Geschwind N. (1978). Anatomical asymmetry as the basis for cerebral dominance. Fed. Proc. 37, 2263–2266 658466

[B57] Geschwind N. Levitsky W. (1968). Human brain: left-right asymmetries in temporal speech region. Science 161, 186–187 10.1126/science.161.3837.186 5657070

[B58] Gibson A. Baker J. Mower G. Glickstein M. (1978). Corticopontine cells in area 18 of the cat. J. Neurophysiol. 41, 484–495 650278 10.1152/jn.1978.41.2.484

[B59] Gibson A. R. Horn K. M. Pong M. (2004). Activation of climbing fibers. Cerebellum 3, 212–221 10.1080/14734220410018995 15686099

[B60] Gilbert P. F. Thach W. T. (1977). Purkinje cell activity during motor learning. Brain Res. 128, 309–328 10.1016/0006-8993(77)90997-0 194656

[B61] Glickstein M. (1969). Organization of the visual pathways. Science 164, 917–926 10.1126/science.164.3882.917 4976681

[B62] Glickstein M. (1993). Motor skills but not cognitive tasks. Trends Neurosci. 16, 450–451 10.1016/0166-2236(93)90074-V 7507616

[B63] Glickstein M. (2007). What does the cerebellum really do? Curr. Biol. 17, R824–R827 10.1016/j.cub.2007.08.009 17925205

[B64] Glickstein M. Cohen J. L. Dixon B. Gibson A. Hollins M. Labossiere E. (1980). Corticopontine visual projections in macaque monkeys. J. Comp. Neurol. 190, 209–229 10.1002/cne.901900202 7381057

[B65] Glickstein M. Gerrits N. Kralj-Hans I. Mercier B. Stein J. Voogd J. (1994). Visual pontocerebellar projections in the macaque. J. Comp. Neurol. 349, 51–72 10.1002/cne.903490105 7852626

[B66] Glickstein M. May J. Mercier B. (1990). Visual corticopontine and tectopontine projections in the macaque. Arch. Ital. Biol. 128, 273–293 1702611

[B67] Glickstein M. May J. G. III Mercier B. E. (1985). Corticopontine projection in the macaque: the distribution of labelled cortical cells after large injections of horseradish peroxidase in the pontine nuclei. J. Comp. Neurol. 235, 343–359 10.1002/cne.902350306 3998215

[B68] Glickstein M. Strata P. Voogd J. (2009a). Cerebellum: history. Neuroscience 162, 549–559 10.1016/j.neuroscience.2009.02.054 19272426

[B69] Glickstein M. Sultan F. Jayakumar A. Voogd J. Hamodeh S. Baizer J. S. (2009b). “Quantification and 3D reconstruction of the hominoid dentate nucleus,” in Neuroscience Meeting Planner (Chicago, IL: Society for Neuroscience), Program No. 460.1.

[B70] Glickstein M. Waller J. Baizer J. S. Brown B. Timmann D. (2005). Cerebellum lesions and finger use. Cerebellum 4, 189–197 10.1080/14734220500201627 16147951

[B71] Goto N. Kakimi S. Kaneko M. (1988). Olivary enlargement: stage of initial astrocytic changes. Clin. Neuropathol. 7, 39–43 3370862

[B72] Hammond G. (2002). Correlates of human handedness in primary motor cortex: a review and hypothesis. Neurosci. Biobehav. Rev. 26, 285–292 10.1016/S0149-7634(02)00003-9 12034131

[B73] Herculano-Houzel S. (2010). Coordinated scaling of cortical and cerebellar numbers of neurons. Front. Neuroanat. 4:12 10.3389/fnana.2010.00012 20300467 PMC2839851

[B74] Hof P. R. Morrison J. H. (1995). Neurofilament protein defines regional patterns of cortical organization in the macaque monkey visual system: a quantitative immunohistochemical analysis. J. Comp. Neurol. 352, 161–186 10.1002/cne.903520202 7721988

[B75] Holloway R. L. de Lacoste M. C. (1986). Sexual dimorphism in the human corpus callosum: an extension and replication study. Hum. Neurobiol. 5, 87–91 3733478

[B76] Hoover J. E. Strick P. L. (1999). The organization of cerebellar and basal ganglia outputs to primary motor cortex as revealed by retrograde transneuronal transport of herpes simplex virus type 1. J. Neurosci. 19, 1446–1463 9952421 10.1523/JNEUROSCI.19-04-01446.1999 PMC6786031

[B77] Horn A. Leigh R. (2011). “The anatomy and physiology of the ocular motor system,” in Handbook of Clinical Neurology, eds Kennard C. Leight R. (Amsterdam: Elsevier), 21–56 10.1016/B978-0-444-52903-9.00008-X 21601062

[B78] Hund-Georgiadis M. Lex U. Friederici A. D. Von Cramon D. Y. (2002). Non-invasive regime for language lateralization in right- and left-handers by means of functional MRI and dichotic listening. Exp. Brain Res. 145, 166–176 10.1007/s00221-002-1090-0 12110956

[B79] Jancke L. Schlaug G. Huang Y. Steinmetz H. (1994). Asymmetry of the planum parietale. Neuroreport 5, 1161–1163 10.1097/00001756-199405000-00035 8080979

[B80] Jones E. G. Dell’Anna M. E. Molinari M. Rausell E. Hashikawa T. (1995). Subdivisions of macaque monkey auditory cortex revealed by calcium-binding protein immunoreactivity. J. Comp. Neurol. 362, 153–170 10.1002/cne.903620202 8576431

[B81] Kaneko C. R. (1992). Effects of ibotenic acid lesions of nucleus prepositus hypoglossi on optokinetic and vestibular eye movements in the alert, trained monkey. Ann. N. Y. Acad. Sci. 656, 408–427 10.1111/j.1749-6632.1992.tb25225.x 1599159

[B82] Kaneko C. R. (1997). Eye movement deficits after ibotenic acid lesions of the nucleus prepositus hypoglossi in monkeys. I. Saccades and fixation. J. Neurophysiol. 78, 1753–1768 9325345 10.1152/jn.1997.78.4.1753

[B83] Kaneko C. R. (1999). Eye movement deficits following ibotenic acid lesions of the nucleus prepositus hypoglossi in monkeys II. Pursuit, vestibular, and optokinetic responses. J. Neurophysiol. 81, 668–681 10036269 10.1152/jn.1999.81.2.668

[B84] Kaneko C. R. Fuchs A. F. (1991). Saccadic eye movement deficits following ibotenic acid lesions of the nuclei raphe interpositus and prepositus hypoglossi in monkey. Acta Otolaryngol. Suppl. 481, 213–215 10.3109/00016489109131383 1927378

[B85] Kelly R. M. Strick P. L. (2003). Cerebellar loops with motor cortex and prefrontal cortex of a nonhuman primate. J. Neurosci. 23, 8432–8444 12968006 10.1523/JNEUROSCI.23-23-08432.2003 PMC6740694

[B86] Kim S. G. Ugurbil K. Strick P. L. (1994). Activation of a cerebellar output nucleus during cognitive processing. Science 265, 949–951 10.1126/science.8052851 8052851

[B87] Kitajima M. Korogi Y. Shimomura O. Sakamoto Y. Hirai T. Miyayama H. (1994). Hypertrophic olivary degeneration: MR imaging and pathologic findings. Radiology 192, 539–543 8029428 10.1148/radiology.192.2.8029428

[B88] Knecht S. Deppe M. Drager B. Bobe L. Lohmann H. Ringelstein E. (2000). Language lateralization in healthy right-handers. Brain 123(Pt 1), 74–81 10.1093/brain/123.1.74 10611122

[B89] Kooy F. (1917). The inferior olive of vertebrates. Folia Neurobiol. 10, 205–369

[B90] Krings T. Foltys H. Meister I. G. Reul J. (2003). Hypertrophic olivary degeneration following pontine haemorrhage: hypertensive crisis or cavernous haemangioma bleeding? J. Neurol. Neurosurg. Psychiatry 74, 797–799 10.1136/jnnp.74.6.797 12754356 PMC1738477

[B91] Kuypers H. G. Lawrence D. G. (1967). Cortical projections to the red nucleus and the brain stem in the rhesus monkey. Brain Res. 4, 151–188 10.1016/0006-8993(67)90004-2 4961812

[B92] LaBossiere E. Glickstein M. (1976). Histological Processing for the Neural Sciences. Springfield, IL: Charles C. Thomas

[B93] Langer T. Fuchs A. F. Chubb M. C. Scudder C. A. Lisberger S. G. (1985). Floccular efferents in the rhesus macaque as revealed by autoradiography and horseradish peroxidase. J. Comp. Neurol. 235, 26–37 10.1002/cne.902350102 3989003

[B94] Le Bihan D. Mangin J. F. Poupon C. Clark C. A. Pappata S. Molko N. (2001). Diffusion tensor imaging: concepts and applications. J. Magn. Reson. Imaging 13, 534–546 10.1002/jmri.1076 11276097

[B95] Leergaard T. B. Lillehaug S. De Schutter E. Bower J. M. Bjaalie J. G. (2006). Topographical organization of pathways from somatosensory cortex through the pontine nuclei to tactile regions of the rat cerebellar hemispheres. Eur. J. Neurosci. 24, 2801–2812 10.1111/j.1460-9568.2006.05150.x 17156205

[B96] Lehman R. A. (1978). The handedness of rhesus monkeys – I. Distribution. Neuropsychologia 16, 33–42 10.1016/0028-3932(78)90040-4 416370

[B97] Levay S. (1991). Response. Science 254, 630 10.1126/science.254.5032.630-b 17774775

[B98] MacLean P. D. (1990). The Triune Brain in Evolution: Role in Paleocerebral Functions. New York: Plenum Press 10.1126/science.250.4978.303-a 17797318

[B99] MacLeod C. E. Zilles K. Schleicher A. Rilling J. K. Gibson K. R. (2003). Expansion of the neocerebellum in *Hominoidea*. J. Hum. Evol. 44, 401–429 10.1016/S0047-2484(03)00028-9 12727461

[B100] Madigan J. Carpenter M. (1971). Cerebellum of the Rhesus Monkey. Baltimore, MD: University Park Press

[B101] Mai J. Rgen K. Paxinos G. Voss T. (2008). Atlas of the Human Brain. Boston: Elsevier/Academic Press

[B102] Marden F. A. (2013). Hypertrophic olivary degeneration due to pontine hemorrhage. JAMA Neurol. 70, 1330 10.1001/2013.jamaneurol.359 23921433

[B103] Matheson A. J. Darlington C. L. Smith P. F. (1999). Further evidence for age-related deficits in human postural function. J. Vestib. Res. 9, 261–264 10472038

[B104] McCormick C. M. Witelson S. F. Kingstone E. (1990). Left-handedness in homosexual men and women: neuroendocrine implications. Psychoneuroendocrinology 15, 69–76 10.1016/0306-4530(90)90048-E 2367617

[B105] Middleton F. A. Strick P. L. (1994). Anatomical evidence for cerebellar and basal ganglia involvement in higher cognitive function. Science 266, 458–461 10.1126/science.7939688 7939688

[B106] Middleton F. A. Strick P. L. (1996). Basal ganglia and cerebellar output influences non-motor function. Mol. Psychiatry 1, 429–433 9154241

[B107] Middleton F. A. Strick P. L. (1997a). Cerebellar output channels. Int. Rev. Neurobiol. 41, 61–82 10.1016/S0074-7742(08)60347-5 9378611

[B108] Middleton F. A. Strick P. L. (1997b). Dentate output channels: motor and cognitive components. Prog. Brain Res. 114, 553–566 10.1016/S0079-6123(08)63386-5 9193166

[B109] Middleton F. A. Strick P. L. (1998). Cerebellar output: motor and cognitive channels. Trends Cogn. Sci. 2, 348–354 10.1016/S1364-6613(98)01220-0 21227231

[B110] Middleton F. A. Strick P. L. (2000). Basal ganglia and cerebellar loops: motor and cognitive circuits. Brain Res. Brain Res. Rev. 31, 236–250 10.1016/S0165-0173(99)00040-5 10719151

[B111] Middleton F. A. Strick P. L. (2001). Cerebellar projections to the prefrontal cortex of the primate. J. Neurosci. 21, 700–712 11160449 10.1523/JNEUROSCI.21-02-00700.2001 PMC6763818

[B112] Molinari M. Dell’Anna M. E. Rausell E. Leggio M. G. Hashikawa T. Jones E. G. (1995). Auditory thalamocortical pathways defined in monkeys by calcium-binding protein immunoreactivity. J. Comp. Neurol. 362, 171–194 10.1002/cne.903620203 8576432

[B113] Moroni M. Veronese S. Benvenuti S. Marrapese G. Sartore-Bianchi A. Di Nicolantonio F. (2005). Gene copy number for epidermal growth factor receptor (EGFR) and clinical response to antiEGFR treatment in colorectal cancer: a cohort study. Lancet Oncol. 6, 279–286 10.1016/S1470-2045(05)70102-9 15863375

[B114] Morton S. M. Bastian A. J. (2004). Cerebellar control of balance and locomotion. Neuroscientist 10, 247–259 10.1177/1073858404263517 15155063

[B115] Olszewski J. Baxter D. (1954). Cytoarchitecture of the Human Brain Stem. Basel: Karger

[B116] Orioli P. J. Strick P. L. (1989). Cerebellar connections with the motor cortex and the arcuate premotor area: an analysis employing retrograde transneuronal transport of WGA-HRP. J. Comp. Neurol. 288, 612–626 10.1002/cne.902880408 2478593

[B117] Paxinos G. (1999). Chemoarchitectonic Atlas of the Rat Forebrain. San Diego: Academic Press

[B118] Paxinos G. Carrive P. Wang H. Wang P.-Y. (1999). Chemoarchitectonic Atlas of the Rat Brainstem. San Diego: Academic Press

[B119] Paxinos G. Huang X. F. (1995). Atlas of the Human Brainstem. San Diego: Academic Press

[B120] Paxinos G. Huang X. F. Toga A. W. (2000). The Rhesus Monkey Brain in Stereotaxic Coordinates. San Diego: Academic Press

[B121] Petersen S. E. Fox P. T. Snyder A. Z. Raichle M. E. (1990). Activation of extrastriate and frontal cortical areas by visual words and word-like stimuli. Science 249, 1041–1044 10.1126/science.2396097 2396097

[B122] Phillips K. A. Sherwood C. C. (2005). Primary motor cortex asymmetry is correlated with handedness in capuchin monkeys (*Cebus apella*). Behav. Neurosci. 119, 1701–1704 10.1037/0735-7044.119.6.1701 16420175

[B123] Pimenta A. F. Strick P. L. Levitt P. (2001). Novel proteoglycan epitope expressed in functionally discrete patterns in primate cortical and subcortical regions. J. Comp. Neurol. 430, 369–388 10.1002/1096-9861(20010212)430:3<369::AID-CNE1037>3.3.CO;2-3 11169474

[B124] Polyak S. L. (1957). The Vertebrate Visual System: Its Origin, Structure, and Function and its Manifestations in Disease with an Analysis of its Role in the Life of Animals and in the Origin of Man, Preceded by a Historical Review of Investigations of the Eye, and of the Visual Pathways and Centers of the Brain. Chicago: University of Chicago Press

[B125] Pujol J. Deus J. Losilla J. M. Capdevila A. (1999). Cerebral lateralization of language in normal left-handed people studied by functional MRI. Neurology 52, 1038–1043 10.1212/WNL.52.5.1038 10102425

[B126] Rausell E. Bae C. S. Vinuela A. Huntley G. W. Jones E. G. (1992a). Calbindin and parvalbumin cells in monkey VPL thalamic nucleus: distribution, laminar cortical projections, and relations to spinothalamic terminations. J. Neurosci. 12, 4088–4111 1328563 10.1523/JNEUROSCI.12-10-04088.1992 PMC6575950

[B127] Rausell E. Cusick C. G. Taub E. Jones E. G. (1992b). Chronic deafferentation in monkeys differentially affects nociceptive and nonnociceptive pathways distinguished by specific calcium-binding proteins and down-regulates gamma-aminobutyric acid type A receptors at thalamic levels. Proc. Natl. Acad. Sci. U.S.A. 89, 2571–2575 10.1073/pnas.89.7.2571 1313562 PMC48703

[B128] Ruigrok T. J. Voogd J. (2000). Organization of projections from the inferior olive to the cerebellar nuclei in the rat. J. Comp. Neurol. 426, 209–228 10.1002/1096-9861(20001016)426:2<209::AID-CNE4>3.0.CO;2-0 10982464

[B129] Sagan C. (1977). The Dragons of Eden: Speculations on the Evolution of Human Intelligence. New York: Random House

[B130] Sartore-Bianchi A. Moroni M. Veronese S. Carnaghi C. Bajetta E. Luppi G. (2007). Epidermal growth factor receptor gene copy number and clinical outcome of metastatic colorectal cancer treated with panitumumab. J. Clin. Oncol. 25, 3238–3245 10.1200/JCO.2007.11.5956 17664472

[B131] Schmahmann J. D. (1998). Dysmetria of thought: clinical consequences of cerebellar dysfunction on cognition and affect. Trends Cogn. Sci. 2, 362–371 10.1016/S1364-6613(98)01218-2 21227233

[B132] Schmahmann J. D. Sherman J. C. (1998). The cerebellar cognitive affective syndrome. Brain 121(Pt 4), 561–579 10.1093/brain/121.4.561 9577385

[B133] Shaywitz B. A. Shaywitz S. E. Pugh K. R. Constable R. T. Skudlarski P. Fulbright R. K. (1995). Sex differences in the functional organization of the brain for language. Nature 373, 607–609 10.1038/373607a0 7854416

[B134] Steinmetz H. (1996). Structure, functional and cerebral asymmetry: in vivo morphometry of the planum temporale. Neurosci. Biobehav. Rev. 20, 587–591 10.1016/0149-7634(95)00071-2 8994197

[B135] Steinmetz H. Rademacher J. Huang Y. X. Hefter H. Zilles K. Thron A. (1989). Cerebral asymmetry: MR planimetry of the human planum temporale. J. Comput. Assist. Tomogr. 13, 996–1005 10.1097/00004728-198911000-00011 2584512

[B136] Stryker M. Blakemore C. (1972). Saccadic and disjunctive eye movements in cats. Vision Res. 12, 2005–2013 10.1016/0042-6989(72)90054-5 4636124

[B137] Sultan F. Braitenberg V. (1993). Shapes and sizes of different mammalian cerebella. A study in quantitative comparative neuroanatomy. J. Hirnforsch. 34, 79–92 8376757

[B138] Sultan F. Glickstein M. (2007). The cerebellum: comparative and animal studies. Cerebellum 6, 168–176 10.1080/14734220701332486 17786812

[B139] Sultan F. Hamodeh S. Baizer J. S. (2010). The human dentate nucleus: a complex shape untangled. Neuroscience 167, 965–968 10.1016/j.neuroscience.2010.03.007 20223281

[B140] Suzuki L. Coulon P. Sabel-Goedknegt E. H. Ruigrok T. J. (2012). Organization of cerebral projections to identified cerebellar zones in the posterior cerebellum of the rat. J. Neurosci. 32, 10854–10869 10.1523/JNEUROSCI.0857-12.2012 22875920 PMC6621006

[B141] Talairach J. Tournoux P. (1988). Co-Planar Stereotaxic Atlas of the Human Brain: 3-Dimensional Proportional System: An Approach to Cerebral Imaging. New York: Thieme Medical Publishers

[B142] Thangnipon W. Taxt T. Brodal P. Storm-Mathisen J. (1983). The corticopontine projection: axotomy-induced loss of high affinity L-glutamate and D-aspartate uptake, but not of gamma-aminobutyrate uptake, glutamate decarboxylase or choline acetyltransferase, in the pontine nuclei. Neuroscience 8, 449–457 10.1016/0306-4522(83)90191-4 6304568

[B143] Tzourio-Mazoyer N. Landeau B. Papathanassiou D. Crivello F. Etard O. Delcroix N. (2002). Automated anatomical labeling of activations in SPM using a macroscopic anatomical parcellation of the MNI MRI single-subject brain. Neuroimage 15, 273–289 10.1006/nimg.2001.0978 11771995

[B144] Van Der Gucht E. Youakim M. Arckens L. Hof P. R. Baizer J. S. (2006). Variations in the structure of the prelunate gyrus in old world monkeys. Anat. Rec. A Discov. Mol. Cell. Evol. Biol. 288, 753–775 10.1002/ar.a.20350 16779809 PMC2837282

[B145] van’t Veer L. J. Dai H. Van De Vijver M. J. He Y. D. Hart A. A. Mao M. (2002). Gene expression profiling predicts clinical outcome of breast cancer. Nature 415, 530–536 10.1038/415530a 11823860

[B146] Voogd J. Glickstein M. (1998a). The anatomy of the cerebellum. Trends Neurosci. 21, 370–375 10.1016/S0166-2236(98)01318-6 9735944

[B147] Voogd J. Glickstein M. (1998b). The anatomy of the cerebellum. Trends Cogn. Sci. 2, 307–313 10.1016/S1364-6613(98)01210-8 21227226

[B148] Westbury C. F. Zatorre R. J. Evans A. C. (1999). Quantifying variability in the planum temporale: a probability map. Cereb. Cortex 9, 392–405 10.1093/cercor/9.4.392 10426418

[B149] Witelson S. F. (1989). Hand and sex differences in the isthmus and genu of the human corpus callosum. A postmortem morphological study. Brain 112(Pt 3), 799–835 10.1093/brain/112.3.799 2731030

[B150] Witelson S. F. (1991). Neural sexual mosaicism: sexual differentiation of the human temporo-parietal region for functional asymmetry. Psychoneuroendocrinology 16, 131–153 10.1016/0306-4530(91)90075-5 1961836

[B151] Witelson S. F. Glezer I. I. Kigar D. L. (1995). Women have greater density of neurons in posterior temporal cortex. J. Neurosci. 15, 3418–3428 7751921 10.1523/JNEUROSCI.15-05-03418.1995 PMC6578233

[B152] Witelson S. F. Goldsmith C. H. (1991). The relationship of hand preference to anatomy of the corpus callosum in men. Brain Res. 545, 175–182 10.1016/0006-8993(91)91284-8 1860044

[B153] Witelson S. F. Kigar D. L. (1992). Sylvian fissure morphology and asymmetry in men and women: bilateral differences in relation to handedness in men. J. Comp. Neurol. 323, 326–340 10.1002/cne.903230303 1460106

[B154] Witelson S. F. Kigar D. L. Harvey T. (1999). The exceptional brain of Albert Einstein. Lancet 353, 2149–2153 10.1016/S0140-6736(98)10327-6 10382713

[B155] Witelson S. F. McCulloch P. B. (1991). Premortem and postmortem measurement to study structure with function: a human brain collection. Schizophr. Bull. 17, 583–591 10.1093/schbul/17.4.583 1805351

[B156] Yang Y. Lisberger S. G. (2013). Duration of Complex Spikes Grades Single-Trial Plasticity and Learning. San Diego, CA: Society for Neuroscience, Program #469.13/FFF17

[B157] Zatorre R. J. Evans A. C. Meyer E. (1994). Neural mechanisms underlying melodic perception and memory for pitch. J. Neurosci. 14, 1908–1919 8158246 10.1523/JNEUROSCI.14-04-01908.1994 PMC6577137

